# Analysis of the minutes of rondas campesinas using text mining techniques

**DOI:** 10.3389/fsoc.2026.1874323

**Published:** 2026-09-09

**Authors:** Lenin Quiñones Huatangari, Raúl Díaz Cabrera, Nicanor Alvarado Carrasco, Frank Fernández Rosillo, Jorge Luis Maicelo Guevara, Omer Cruz Caro, Segundo Salazar Serván

**Affiliations:** 1 Universidad Nacional Toribio Rodríguez de Mendoza de Amazonas, Facultad de Ingeniería Zootecnista y Biotecnología, Escuela Profesional de Ingeniería de Ciencia de Datos e Inteligencia Artificial, Chachapoyas, Peru; 2 Instituto de Educación Superior Privado “CERTUS”, Carrera de Diseño y Desarrollo de Software, Lima, Peru; 3 Universidad Nacional de Jaén, Instituto de Investigación en Ciencias Sociales, Jaén, Cajamarca, Peru; 4 Universidad Nacional de Jaén, Instituto de Investigación en Ciencia de Datos, Jaén, Cajamarca, Peru; 5 Pontificia Universidad Católica del Perú, Escuela Profesional de Economía, Lima, Peru; 6 Universidad Nacional Toribio Rodríguez de Mendoza de Amazonas, Oficina de Gestión de la Calidad, Chachapoyas, Peru; 7 Universidad Nacional Toribio Rodríguez de Mendoza de Amazonas, Dirección de Admisión y Asuntos Académicos, Chachapoyas, Peru

**Keywords:** artificial intelligence, governance, intercultural approach, legal pluralism, topical analysis

## Abstract

The rondas campesinas are rural community justice organizations that emerged among impoverished indigenous communities in the Andes and are the driving force behind the largest and most influential grassroots movement in north-eastern Peru. The aim of the research was to unveil the characteristics, thematic approaches and working dynamics of the rondas campesinas using text mining as a technique for information analysis. Seventy-four minutes from Huaquillas, Namballe and San Jose de Lourdes in the province of San Ignacio, department of Cajamarca were analyzed, covering the period from 2012 to 2024. The analysis included five steps: data collection and pre-processing by eliminating stopwords and tokenization, keyword identification using the word cloud technique, word quantification using the Term Frequency-Inverse Document Frequency method, latent topic analysis through Latent Dirichlet assignment and vector representation through modeling with Word2Vec. These steps allowed the identification of community problems, social control actions and emerging trends in community organization. The text mining technique is a tool to analyze minutes not only of rondas campesinas but also of organizations or institutions that want to know with certainty the thematic approaches they develop.

## 1 Introduction

Throughout the history of the formation of civilizations around the world, various mechanisms of social dynamics have been observed, circumscribed by the search for the common good as a primary goal (Touraine, 1985), has led to the grouping of individuals around ideas, philosophies and premises of collective interest (Edelman, 2001), that have governed the consensus and concertation of nations, which has contributed to the consolidation of modern societies (Melucci, 1984). Therefore, there are own or external factors that bring people together, territorially or socially, with common rather than individual objectives, interests and rules (Lázaro Aquino, 2024). This social dynamism due to the inherent need for development and growth to satisfy the lifestyle of citizens causes political, geopolitical and technological phenomena, which in some cases contradict an established order, violating common law and prioritizing supra-individual interests (Hamel and Maheu, 2001); where organized civil society seeks and finds new mechanisms that contribute to being an effective alternative in established struggles (Brisman et al., 2016). This is why social movements develop under different geographical, political and situational circumstances, where the search for equity and justice of the interest group or civil society takes precedence and converges (Giugni, 1999). This constitutes a challenge in the study, understanding and addressing of social conflicts based on historical and current data (Doerr et al., 2013), where new technological trends (Rezaev and Tregubova, 2018) contribute to and play an important role in processing and searching for recurring patterns or behaviors in these events (Olsher, 2015).

In South America, social movements and the development of collectives are the product of political phenomena with a lack of precision and effectiveness in decision-making on issues that concern societies (Inclán, 2018). Rural social movements develop in contexts of historical inequalities, territorial exclusion, and asymmetrical relationships between communities and states (Lopez-Flores, 2024). From this perspective, internal colonialism helps explain why methods of domination over indigenous and rural communities still exist within nations (Casanova, 1965). On the other hand, the coloniality of power explains why social, political, and economic hierarchies inherited from the colonial order persist (Quijano, 2000). Furthermore, the physical and administrative distance between rural communities and government institutions fosters the development of methods of collective collaboration (Davis, 1999). In this context, the “rondas campesinas” emerge as expressions of community self-management related to territorial protection, social regulation, and the building of collective consensus.

On 27 April 1990, peasants from northern Peru stormed the streets of Chota, department of Cajamarca, with the intention of creating other forms of non-state power and justice (Acuña, 2019). The rondas campesinas are rural organizations formed in Cajamarca (Chillihuani Ttito, 2020) of community justice (Nuñez Palomino, 1996), a mass movement of night watchman patrols emerged among impoverished indigenous communities in the Andes (Escalante, 2020); driving the largest and most influential grassroots movement in north-eastern Peru (Shanee, 2019). The minutes of rondas campesinas are important documents that serve as instruments of legitimacy, transparency and collective memory. Therefore, the Peruvian state recognizes them as organizations that can support rural communities (Piccoli, 2009). Plenary Agreement N° 1/2009/CJ-116 is a contribution of the Supreme Court of Peru that contains normative elements to concretize the coordination between the special communal jurisdiction and the ordinary jurisdiction, given the constitutional and conventional mandate that sustains the recognition of the jurisdictional function of the peasant patrols in Peru (Alarcón-Requejo, 2023).

The production, modification and transformation of data are the result of scientific and technical processes, reflecting human values and social relations (Kitchin, 2021). Data have been used as tools and weapons to argue for what is true (Wiggins and Jones, 2023). However, technology has driven their mass creation (Yamin, 2019), facilitating storage (Feldman and Sanger, 2006) and becoming complex (Sawitri, 2024); more than 80% of them are unstructured or semi-structured (Talib et al., 2016) and are in text form (Patel, 2017). It is therefore necessary to extract value and knowledge from these data sets (Elgendy and Elragal, 2014). Advances in the fields of mathematics, computer science, machine learning and natural language processing can collectively capture, classify and interpret words and their contexts (Xiaojun, 2011).

Text mining (TM) is defined as the process of extracting meaningful information and hidden patterns from large volumes of unstructured textual data (del Castillo Zayas and Leiva Mederos, 2007; Markellos, 2009; Markellos, 2009). Its growth has been driven by technological advances (Zanini and Dhawan, 2015), there is software available to employ TM (Percha, 2021), some of the open source tools: KNIME, RapidMiner, Weka, R, and Python (Hofmann and Chisholm, 2016). TM has a range of applications as an information analysis technique (Qamar and Raza, 2024): It reveals the characteristics, thematic focus and working dynamics of a research group (Pérez and Reyes, 2022), understands how citizens perceive climate change and how it impacts on the urban environment and changes the landscape (Choi et al., 2022), recognizes, defines and selects problems (Gyódi et al., 2023), generates a methodology for quantifying socio-political conflicts related to natural resource exploitation through newspaper reports (Palazzo, 2017), assesses perceptions of the environmental, social and governance visions of companies in line with the Sustainable Development Goals (Yoon et al., 2023), analyses news from the Russian news agency RIA Novosti and the Ukrainian news agency during the Russian invasion of Ukraine (Ptaszek et al., 2024), scans data sources for potential threats, trends and accelerates forecasting process (de Boer et al., 2019), extracts health outcomes for the development of prognostic models (Grotenhuis et al., 2024), elucidates the analytical bias of social networking and TM techniques (Singh et al., 2017).

Within this framework, the present study aims to reveal the characteristics, thematic focuses, and organizational dynamics of the rondas campesinas located in northern Peru, through the analysis of their minutes using TM techniques. The study is justified because these minutes are not merely administrative records, but social documents through which communities express agreements, concerns, forms of authority, mechanisms of social control, and claims related to territorial defense, the environment, mining, and relations with local authorities. From the perspective of the sociology of law, analyzing these records makes it possible to understand how rondas campesinas produce and legitimize communal norms, organize collective memory, and sustain their own forms of justice in dialogue or tension with the state legal order. Therefore, the use of tools such as TF-IDF, LDA, and Word2Vec not only provides a methodological strategy for processing unstructured information, but also makes visible discursive patterns that help interpret legal pluralism, territorial governance, and collective action in rural contexts of northern Peru.

## 2 Materials and methods

### 2.1 Research framework

The minutes of rondas campesinas are documentary records that function as instruments of legitimacy, transparency, and collective memory. They register community meetings, issues discussed, interventions, agreements, commitments, and decisions related to local organization, social control, conflict management, authorities, mining, environmental concerns, and territorial defense. Although their structure varies according to locality and year, they usually include a heading, date, place of meeting, identification of the ronda or local base, participants or authorities, agenda, development of the issues discussed, final agreements, and signatures.

In order to analyze the minutes through TM, 74 min were collected from the rondas campesinas of the hamlet of Huaquillas and the districts of Namballe and San José de Lourdes in the province of San Ignacio, department of Cajamarca. The corpus corresponds to the documents provided by the representatives of these organizations and authorized for scanning and analysis, covering the period from 4 March 2012 to 14 March 2024, which provides a broad temporal coverage for an exploratory documentary analysis.

### 2.2 Methodology

**Figure 1:**
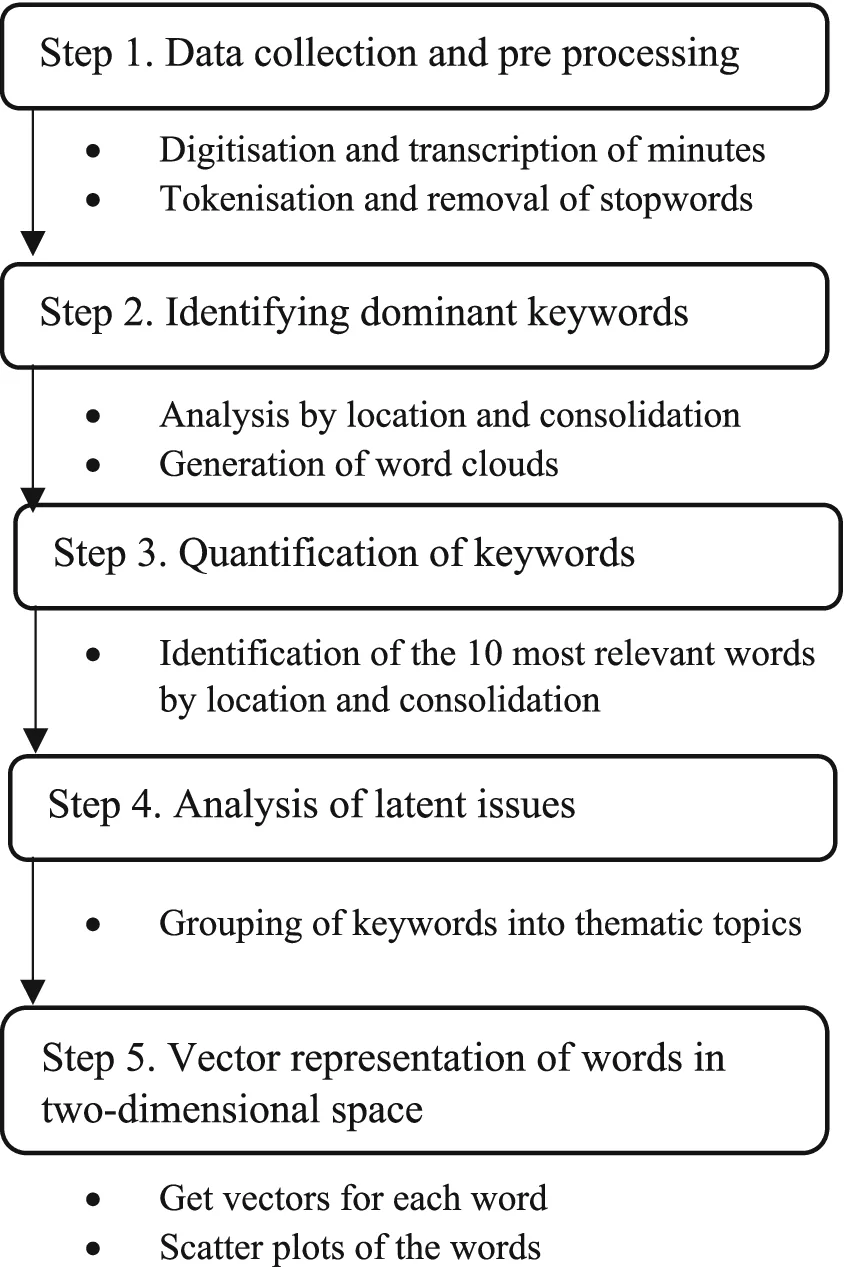
Actions in each of the five steps. The methodological workflow was applied to the original Spanish-language minutes. The source corpus was not translated prior to preprocessing or text-mining analysis.

Figure 1 presents the analysis process in five steps adapted from Han et al. (2024): (1) data collection and pre-processing; (2) identification of dominant keywords through word cloud visualization; (3) keyword quantification using Term Frequency-Inverse Document Frequency (TF-IDF); (4) latent topic analysis using Latent Dirichlet Allocation (LDA); and (5) vector representation through Word2Vec modeling and t-SNE visualization.

#### 2.2.1 Step 1: data collection and pre processing

Copies of the minutes were provided by the heads of the peasant patrols of Huaquillas (20), Namballe (18), and San José de Lourdes (36). They were scanned and transcribed with artificial intelligence (ChatGPT Plus) (Fones and Fones, 2025), then reviewed individually to ensure the correct transcription of the information. The collected data were synthesized into CSV files.

Once the data collection was completed, the cleaning process was carried out, which included the elimination of stopwords, punctuation marks, numerical values, etc., from the data (Sakthi Vel, 2021). This procedure divides the text into smaller units called tokens, generally corresponding to individual words. This stage is fundamental, as it allows for a more precise interpretation and analysis of the information content (Manning et al., 2009). Both tokenization and empty word removal are important processes in text analysis that allow researchers and practitioners to focus on the meaningful words and phrases in textual data (Alaminos-Fernández, 2003).

#### 2.2.2 Step 2: identifying dominant keywords

The word cloud technique was applied individually to the databases obtained from the records of Huaquillas, Namballe, and San José de Lourdes; as well as to the dataset integrating the three localities to visualize and summarize volumes of text, highlighting the most frequent and relevant words (Heimerl et al., 2014). For this purpose, the Python 3.11, wordcloud library was used to display the relevant words in each of the contexts analyzed (Dong et al., 2022).

#### 2.2.3 Step 3: quantification of keywords

The Term Frequency-Inverse Document Frequency (TF-IDF) method was used to calculate the weights of the characteristic words within the text corpus (Kim and Gil, 2019). TF-IDF is a weighted measure to assess the importance of a word in a set of documents, taking into account the frequency of the word in the documents and the rarity of the word in the whole set of documents (Salton, 1983). It facilitates the identification of the most relevant words in the location databases, providing a significant advantage for a better interpretation of the results (Yun-tao et al., 2005). The 10 most relevant words from each database belonging to each site, as well as from the global database integrating the three sites, were ordered in descending order.

#### 2.2.4 Step 4: analysis of latent issues

Latent Dirichlet allocation (LDA) is a generative probabilistic model applied to a corpus (Blei et al., 2003). It consists of representing documents as random mixtures of latent topics, where each topic is characterized by a specific distribution of words (Blei et al., 2003). These thematic clusters allow for a more precise identification of emerging trends in each of the sites analyzed (Soliman et al., 2023). Once the sets of words have been defined, a title is assigned to each topic in order to facilitate their interpretation.

For the LDA analysis, independent models were estimated for each documentary subset: Huaquillas, Namballe, San Jose de Lourdes, and the integrated corpus of the three localities. The number of topics was defined separately for each subset, considering differences in corpus size, lexical composition, and thematic density. Candidate models with different values of k were reviewed, and the final number of topics was selected through qualitative validation based on three criteria: semantic coherence of the most representative words, low redundancy between topics, and interpretability of the resulting clusters in relation to the documentary content of the minutes. Based on this procedure, three topics were retained for Huaquillas, three for Namballe, four for San José de Lourdes, and five for the integrated corpus.

#### 2.2.5 Step 5: vector representation of words in two-dimensional space

The vector representation of words was performed using Word2Vec, a model based on shallow neural networks that generates word vectors from a text corpus (Mikolov et al., 2013). In this study, the embedding process was operationalized at the unigram level. After preprocessing, which included the removal of stopwords, punctuation marks, numerical values, personal names, and non-relevant formal elements, the retained tokens were used to train the Word2Vec model. Bigrams and higher-order n-grams were not included at this stage, since the objective was to examine semantic proximity among individual terms within the minutes.

The model was implemented using the Python gensim library and trained on the documentary corpus from Huaquillas, Namballe, and San José de Lourdes. Parameters such as window size, embedding dimensions, and minimum word frequency were adjusted during model training (Desai et al., 2022). After generating the word embeddings, t-SNE was applied to reduce the vector representations to a two-dimensional space, facilitating visual interpretation (Taskesen and Reinders, 2016). In the resulting maps, each point represents a term retained in the Word2Vec vocabulary after preprocessing, and spatial proximity indicates semantic proximity within the analyzed corpus (Pérez-Serrano et al., 2022). The interpretation focused on terms with greater analytical relevance according to their recurrence, TF-IDF values, and relationship with the LDA topics.

## 3 Results

Taking into account the objective of the study, which was to reveal the characteristics, thematic approaches and work dynamics of the rondas campesinas in three places in the province of San Ignacio in the Department of Cajamarca in Peru, using TM as a technique for analyzing information, the results are presented based on the methodology employed.

**Figure 2:**
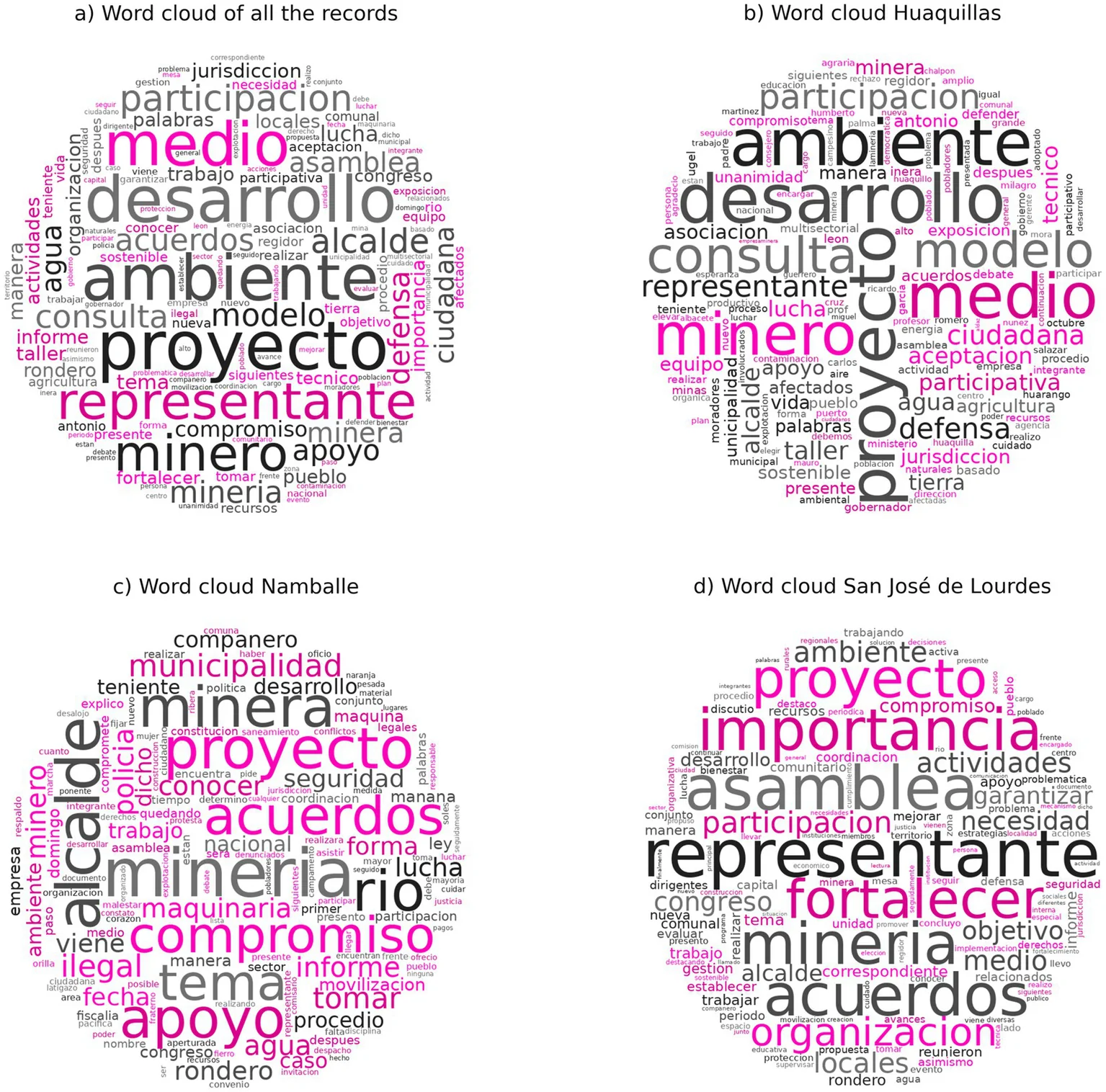
Word clouds of the corpora analyzed by locality: **(a)** Integrated corpus of the three localities; **(b)** Huaquillas; **(c)** Namballe; and **(d)** San José de Lourdes. The word clouds were generated from the original Spanish-language minutes. The terms were not translated prior to analysis in order to preserve their original frequencies and contextual meanings and to avoid potential translation-induced bias.

The word cloud procedure made it possible to identify the most recurrent concepts in the documents examined (Jayashankar and Sridaran, 2017). In the dataset covering the three localities (Figure 2a), the words “project,” “environment,” “development,” “representative,” and “means” stand out, indicating that the topics addressed are related to development initiatives, environmental concerns, and the participation of community stakeholders. In Huaquillas (Figure 2b), terms related to the management of mining projects, consultation processes, and the protection of natural resources stand out, such as “environment,” “development,” “project,” “means,” “consultation,” and “model.” In Namballe (Figure 2c), the words “mining,” “mayor,” “project,” “agreements,” “commitment,” and “support” stand out. This reflects an agenda linked to mining activity. It also reflects coordination with local authorities and the establishment of collective commitments. In San José de Lourdes (Figure 2d), a greater emphasis is observed on the terms “representative,” “importance,” “project,” “assembly,” “strengthen,” “mining,” “agreements,” and “organization.” These terms focus on communal representation, organizational strengthening, and decision-making regarding mining and territorial matters.

Furthermore, keywords were quantified using the TF-IDF technique. As shown in Table 1, the concept “development” obtained the highest weight in Huaquillas, with a value of 0.1409, and in the integrated dataset of the three localities, with 0.0480. In Namballe, the most important word was “mining,” with a value of 0.0702, whereas in San José de Lourdes the word “assembly” stood out, with a value of 0.0563. These results have been complemented by the information presented in the word clouds, which show that discursive priorities vary by locality: “development” and “consultation” in Huaquillas, “mining” and “coordination with authorities” in Namballe, and “assembly-based organization” in San José de Lourdes.

**Table 1:** TF-IDF of the Huaquillas, Namballe, San José de Lourdes and the three locations.

Huaquillas		Namballe		San Jose de Lourdes		Three locations	
Word	TF-IDF	Word	TF-IDF	Word	TF-IDF	Word	TF-IDF
Desarrollo	0.1409	Mineria	0.0702	Asamblea	0.0563	Desarrollo	0.0480
Consulta	0.1172	Alcalde	0.0623	Congreso	0.0494	Ambiente	0.0435
Modelo Desarrollo	0.1094	Ilegal	0.06	Importancia	0.0456	Medio	0.0419
Modelo	0.1050	Minera	0.0596	Organizacion	0.0453	Medio Ambiente	0.0404
Proyecto Minero	0.0933	Rio	0.0581	Ambiente	0.0444	Proyecto	0.0400
Participacion	0.0753	Apoyo	0.0546	Fortalecer	0.0441	Participacion	0.0373
Participacion Ciudadana	0.0726	Municipalidad	0.0541	Proyectos	0.0440	Consulta	0.0373
Consulta Participacion	0.0720	Conocer	0.0515	Mineria	0.0427	Mineria	0.0366
Ciudadana	0.0701	Acuerdos	0.0504	Participacion	0.0421	Modelo Desarrollo	0.0356
Taller	0.0695	Maquinaria	0.0492	Medio Ambiente	0.0411	Modelo	0.0348
Defensa	0.0650	Proyecto	0.0477	Medio	0.0411	Minero	0.0341

TF-IDF values were calculated from the original Spanish-language corpus. Keywords are therefore presented in Spanish, as prior translation could have altered tokenisation, term frequencies, and the resulting relevance weights.

The LDA analysis made it possible to identify five key themes in each of the documents from Huaquillas, Namballe, San José de Lourdes, and the three localities combined. The themes were labeled based on the words with the highest probability of occurrence within each cluster and their coherence with the content of the minutes. Table 2 presents the results and reveals the presence of themes related to mining projects, environmental advocacy, consultation processes, coordination with authorities, the establishment of agreements, and the strengthening of communal organization.

**Table 2:** Suggested topics from the minutes of the rondas campesinas by LDA.

Key words	Suggested topic
Huaquillas	
0.051 “desarrollo” + 0.042 “consulta” + 0.038 “modelo” + 0.030 “minero” + 0.028 “participación”	Participatory consultation and mining development models
0.025 “agua” + 0.025 “defensa” + 0.025 “tierra” + 0.025 “aire” + 0.020 “minero”	Defense of water, land, and air against mining impacts
0.023 “defensa” + 0.021 “asociación” + 0.017 “acuerdos” + 0.017 “minero” + 0.015 “asamblea”	Collective agreements and commitments of mining-affected groups
0.029 “lucha” + 0.021 “defensa” + 0.021 “apoyo” + 0.016 “defender” + 0.015 “alcalde”	Collective struggle and institutional support for environmental defense
0.023 “minero” + 0.019 “desarrollo” + 0.019 “afectados” + 0.016 “asociación” + 0.016 “tierra”	Mining development and protection of affected territories
Namballe	
0.021 “congreso” + 0.019 “maquinaria” + 0.017 “procedió” + 0.016 “máquina” + 0.016 “explicó”	Congress proceedings and machinery management
0.039 “conocer” + 0.039 “compañeros” + 0.033 “minería” + 0.027 “asistir” + 0.014 “policía”	Community participation and commitments regarding mining
0.022 “alcalde” + 0.020 “informe” + 0.016 “desarrollo” + 0.016 “minera” + 0.015 “acuerdos”	Coordination with local authorities on mining and development
0.052 “proyecto” + 0.033 “apoyo” + 0.033 “lucha” + 0.029 “alcalde” + 0.020 “minería”	Collective support and struggle concerning mining projects
0.042 “minería” + 0.038 “río” + 0.034 “ilegal” + 0.022 “acuerdos” + 0.022 “participación”	Mobilization and agreements against illegal mining in rivers
San José de Lourdes	
0.017 “acuerdos” + 0.015 “informe” + 0.013 “ronderos” + 0.012 “congreso” + 0.012 “minería”	Ronda agreements and reports on mining issues
0.020 “actividades” + 0.018 “importancia” + 0.017 “fortalecer” + 0.016 “locales” + 0.011 “comunal”	Strengthening local activities and communal resources
0.019 “importancia” + 0.017 “asamblea” + 0.016 “proyectos” + 0.015 “fortalecer” + 0.014 “gestión”	Assembly management and organizational strengthening
0.020 “minería” + 0.015 “asamblea” + 0.014 “participación” + 0.012 “ambiente” + 0.010 “representante”	Community participation in mining and environmental decisions
0.022 “congreso” + 0.017 “acuerdos” + 0.012 “organización” + 0.010 “mejorar” + 0.010 “fortalecer”	Congresses and commitments for organizational development
Three locations	
0.017 “proyecto” + 0.015 “alcalde” + 0.013 “minería” + 0.012 “lucha” + 0.011 “apoyo”	Mining projects, local authorities, and collective action
0.027 “ambiente” + 0.026 “medio” + 0.017 “minería” + 0.014 “alcalde” + 0.012 “lucha”	Environmental defense in the face of mining activity
0.013 “explicó” + 0.011 “manera” + 0.009 “tema” + 0.007 “ilegal” + 0.007 “río”	Community discussion of illegal mining and river-related problems
0.012 “asamblea” + 0.012 “acuerdos” + 0.012 “organización” + 0.011 “fortalecer” + 0.009 “participación”	Organizational strengthening and collective participation
0.036 “desarrollo” + 0.026 “modelo” + 0.026 “consulta” + 0.025 “proyecto” + 0.025 “minero”	Participatory consultation on mining development projects

The keywords were retained in Spanish because the LDA models were fitted to the original-language corpus. The suggested topic labels were subsequently expressed in English for reporting purposes. No prior translation of the minutes was performed, thereby preserving the original word distributions and thematic structure.

The issues identified reveal disparities in the concerns documented by the rondas campesinas across each jurisdiction. In Huaquillas, the predominant themes are consultation processes, mining development models, and the defense of water, land, and other natural resources. In Namballe, the themes are primarily related to mining activity, coordination with local authorities, and mobilization against illegal mining in the rivers. In San José de Lourdes, assemblies, agreements, project management, and the strengthening of communal organization take on greater relevance. In the integrated corpus, these contents converge into themes related to environmental defense, collective participation, engagement with authorities, and consultation on mining development projects.

**Figure 3:**
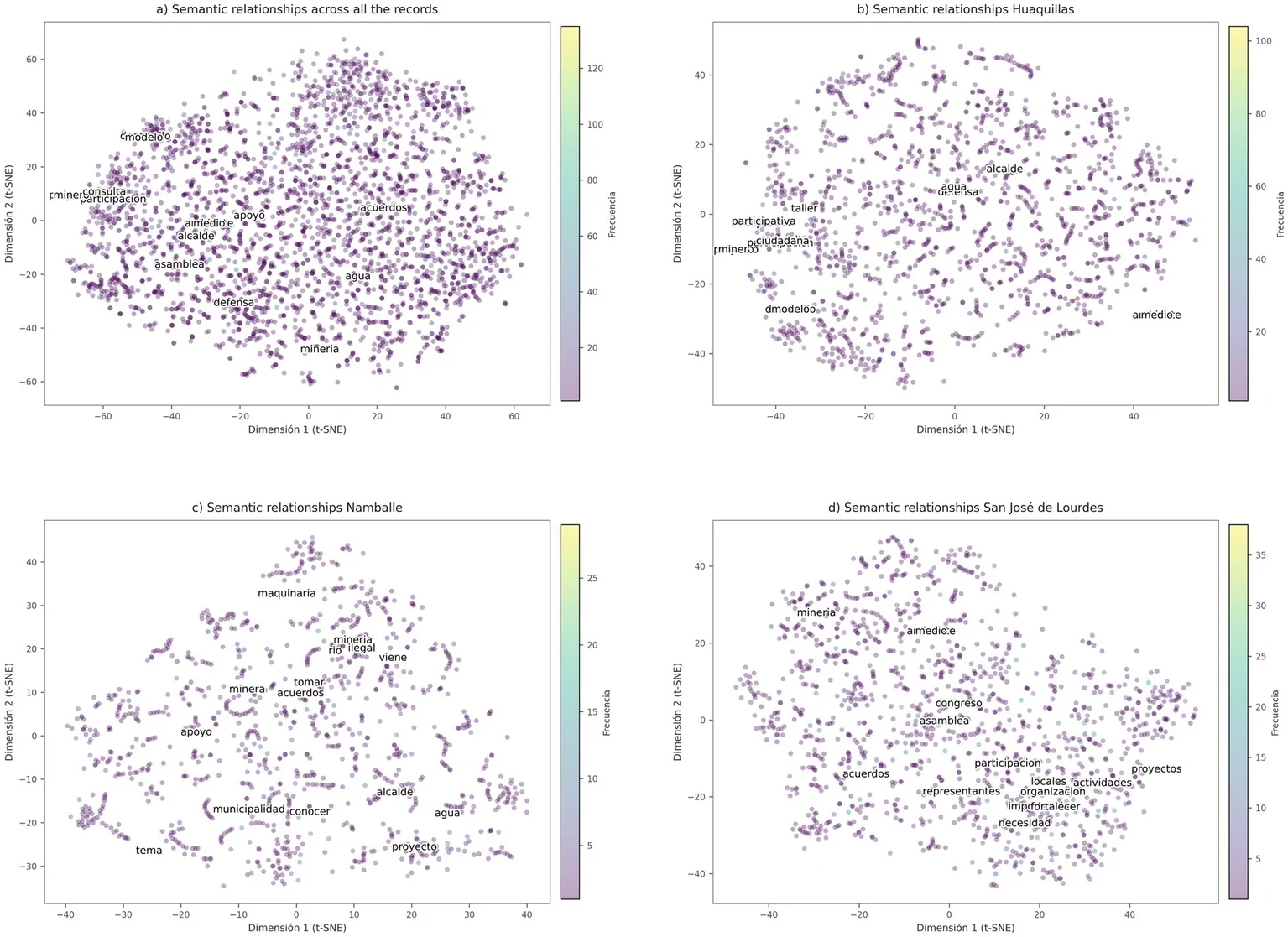
Two-dimensional semantic distribution of the terms present in the minutes of the Rondas Campesinas using Word2Vec and t-SNE: **(a)** Integrated corpus of the three localities; **(b)** Huaquillas; **(c)** Namballe; and **(d)** San José de Lourdes. Word2Vec embeddings and t-SNE projections were generated from the original Spanish-language corpus. The terms were retained in Spanish to preserve their contextual and semantic relationships and to avoid distortions potentially introduced through translation.

To examine the semantic relationships among the terms retained after preprocessing, Word2Vec was used together with the t-SNE technique. Figure 3 shows the distribution of the words in the analyzed dataset in two dimensions. In this representation, the proximity between terms indicates that they tend to be used in similar contexts within the minutes, reflecting a relationship of compatibility and frequency of use. However, due to the characteristics of t-SNE, the interpretation focuses primarily on local clusters rather than on the absolute distances between all points in the space. The highlighted terms were selected based on their recurrence, the values obtained through TF-IDF, and their relationship to the themes identified through LDA.

In the dataset covering the three localities (Figure 3a), a proximity can be observed among the words “consultation,” “participation,” and “mining,” reflecting the connection between participatory processes and the management of mining projects. Furthermore, the words “assembly,” “mayor,” and “environment” are located in a nearby sector, which reflects the involvement of authorities and communal deliberation spaces in environmental matters. The presence of other terms such as “defense,” “mining,” “agreements,” and “water” in different areas of the semantic space demonstrates that the defense of the territory, the use of natural resources, and the adoption of agreements are recurring themes. However, these do not necessarily cluster into a single category.

In Huaquillas (Figure 3b), the words “participatory,” “participation,” “citizen,” “workshop,” and “mining” appear close to one another, suggesting a relationship between community participation, the venues where training is provided, and the manner in which mining activity is conducted. Furthermore, a close relationship is observed between water, defense, and mayor, reflecting the debate over the protection of water resources and the involvement of local authorities. The term “model” appears in another sector of the map, associated with content on development and consultation, which is identified through other analytical techniques.

In Namballe (Figure 3c), a concentration of terms related to mining, the river, and illegality is observed, suggesting the recurrence of issues associated with illegal mining and its potential repercussions for water resources. The terms “mine,” “agreements,” and “take” appear close together, indicating the adoption of decisions regarding this activity, which demands a substantial amount of resources. The positioning of the terms “municipality,” “mayor,” “project,” “support,” and “water” reflects the presence of content related to institutional coordination, local projects, and the protection of natural resources, indicating that these elements are fundamental to the development and effective management of local projects.

In San José de Lourdes (Figure 3d), a clearly identifiable cluster of terms related to the strengthening of communal organization is observed, including “participation,” “representatives,” “local,” “organization,” “activities,” “strengthen,” “importance,” and “need.” The words “congress” and “assembly” appear together in the central area, highlighting the relevance of spaces where people gather to discuss and make decisions. Mining and the environment, by contrast, are located in a different sector.

## 4 Discussion

TM techniques have become increasingly important for analyzing large document collections, particularly those containing unstructured or semi structured information, such as meeting minutes, reports, news articles, institutional records, and community documents (Cogburn et al., 2023; Hassani et al., 2020; Jadhav et al., 2023; Talabis et al., 2015). This study focused on the analysis of a textual corpus consisting of 74 meeting minutes from the rondas campesinas of Huaquillas, Namballe, and San Jose de Lourdes, located in the province of San Ignacio, department of Cajamarca, covering the period from 2012 to 2024. These documents are particularly relevant because the rondas campesinas not only carry out community surveillance and control activities, but also provide spaces for discussing local issues, making collective decisions, establishing community rules, and defending the territory.

The results indicate that the rondas campesinas are no longer confined to the community surveillance activities that originally led to their creation, revealing a significant transformation in their functions and responsibilities. Their minutes record activities such as the administration of justice, conflict resolution, territorial protection, institutional coordination, and participation in decisions affecting the community. This finding is consistent with previous research indicating that these organizations initially emerged in response to cattle theft but gradually expanded their activities to include community justice, social control, and the protection of collective interests (Chillihuani Ttito, 2020; Escalante, 2020; Nuñez Palomino, 1996; Piccoli, 2009).

A noteworthy finding is that the concerns identified are not expressed uniformly across the three localities. The observed differences suggest that each ronda campesina shapes its agenda according to the problems affecting its territory, its organizational experience, and the nature of its relationship with public authorities. In Huaquillas, the prominence of issues related to consultation and development models points to concern over the conditions under which mining projects are proposed and the extent to which local populations participate in decisions that may affect their resources. In Namballe, the relationship among mining, local authorities, and water resources reflects a context more closely associated with the regulation of extractive activities and the pursuit of institutional responses. In San José de Lourdes, the importance attributed to assemblies, representation, and organizational strengthening indicates that the community’s capacity to respond relies primarily on its internal mechanisms of deliberation.

These differences show that rondas campesinas do not operate as homogeneous organizations, despite sharing principles of collective organization, territorial defense, and joint action. Their functioning is shaped by local conditions and specific historical processes, which are, in turn, influenced by social, economic, political, and cultural factors that vary across contexts. This interpretation is consistent with Lázaro Aquino (2024), who argues that collective action in rural communities is constructed around territorial conflicts, shared needs, and particular relationships with state and economic actors. Accordingly, variations between localities should not be understood as inconsistencies, but rather as expressions of the organizational adaptability of the rondas campesinas.

The prominence of mining and environmental issues indicates that rondas campesinas conceive of territory as a social and collective space rather than merely as a source of economic resources. Discussions concerning water, land, the environment, and extractive projects show that territorial defense is closely linked to the continuity of productive activities, community security, and local living conditions. This finding is consistent with Shanee (2019), who identifies the rondas campesinas of northern Peru as key actors in environmental conservation and in the regulation of natural-resource use.

The TF-IDF analysis reveals that the discursive priorities of the rondas campesinas vary according to the characteristics of each territory. In Huaquillas, the relationship among development, consultation, and mining projects indicates that extractive activities are debated in connection with community participation and the preferred development model. In Namballe, the association among illegal mining, rivers, and public authorities reflects a stronger concern with regulating extractive activities and safeguarding water resources. In San José de Lourdes, the prominence of assemblies and organizational strengthening shows that collective deliberation constitutes a central mechanism for addressing local problems. These differences demonstrate that the rondas campesinas do not follow a uniform agenda, but respond to specific social, environmental, and institutional circumstances. This interpretation is consistent with the purpose of TF-IDF, which identifies the terms that distinguish one corpus from others beyond their mere frequency of occurrence (Kim and Gil, 2019; Qaiser and Ali, 2018).

The combined use of word clouds, Word2Vec, and t-SNE made it possible to examine both the predominant concepts and the relationships among them within rondero discourse. Word clouds provided an initial visual overview, whereas vector-based models enabled the identification of semantic associations derived from similar contexts of use (Heimerl et al., 2014; John et al., 2018; Lohmann et al., 2015; Mikolov et al., 2013; Pérez-Serrano et al., 2022). The observed relationships among mining, consultation, participation, water, authorities, and organization suggest that environmental defense is closely integrated with community decision-making and institutional coordination. Accordingly, issues concerning natural resources, territory, and organizational structure do not emerge in isolation, but as interconnected dimensions of the governance practices developed by the rondas campesinas.

Environmental pollution resulting from various productive and extractive activities is a subject of study for the social sciences due to emerging environmental social conflicts (Yearley, 2013), is corroborated by the first topic suggested from the minutes of the rondas campesinas in the three sites by LDA (Table 2). Highlighting the use of TM to study environmental conflicts, such as the regional ones in South Korea (Lee and Kim, 2020) and to measure the number of social conflicts related to the exploitation of non-renewable natural resources (Albrieu and Palazzo, 2020). These findings confirm that members of the rondas campesinas take on dynamic roles as leaders and initiators of conservation efforts in their local area, including the implementation of natural resource use control, extractive operations and environmental education (Shanee, 2019).

The findings generated through the different text-mining techniques proved to be mutually complementary, allowing the corpus to be examined from several analytical perspectives. Word clouds identified recurrent terms, TF-IDF distinguished the words that characterized each locality, LDA revealed underlying thematic structures, and Word2Vec combined with t-SNE represented semantic relationships among concepts used in similar contexts. Consequently, variations across the results should not be regarded as contradictions, but rather as distinct approaches to interpreting the discourse contained in the minutes. The integration of these techniques strengthens the analysis of large and unstructured corpora, particularly in research addressing social, environmental, and institutional issues (Chandran et al., 2025; Curiskis et al., 2020; Hofmann and Chisholm, 2016; Kataishi, 2026; Qamar and Raza, 2024).

From a sociological perspective, the findings indicate that the minutes do more than document agreements; they also reflect practices of communal governance, collective deliberation, coordination with public authorities, and the protection of shared resources. The presence of content related to assemblies, participation, agreements, mining, the environment, and authorities demonstrates that the rondas campesinas formulate organized responses to territorial problems. This dynamic is consistent with their recognition as organizations that perform functions of community justice and social coordination in areas where state presence remains limited or fragmented (Alarcón-Requejo, 2023; Nuñez Palomino, 1996; Piccoli, 2009). These findings may also be interpreted through the lens of legal pluralism, since the communal decisions recorded in the minutes coexist, interact, or come into tension with the state legal order within historical relationships shaped by territorial inequalities and persistent structures of power (Alarcón-Requejo, 2023; Casanova, 1965; Quijano, 2000).

TM techniques make it possible to identify lexical recurrences, patterns of association, and thematic clusters within a broad documentary corpus; however, these results do not, by themselves, constitute a complete sociological explanation. Their analytical value depends on the ability to situate this evidence within the historical, territorial, and institutional context in which the rondas campesinas operate. In this regard, recurring terms such as environment, mining, authorities, federation, and communities acquire deeper meaning when they are connected to the organizational trajectory of these institutions, their relationship with the State, the socio-environmental tensions present in the territory, and communal forms of deliberation, social control, and normative production. Therefore, the central contribution of this study does not lie solely in the application of computational tools to the analysis of community documents, but in demonstrating that these techniques can broaden the understanding of rural institutions of justice and governance when they are articulated with a sociological, contextual, critical, and intercultural interpretation.

Finally, the study has several limitations that should be considered when interpreting the findings. First, the analysis was based exclusively on 74 meeting minutes from three localities in the province of San Ignacio. Therefore, the results cannot be generalized to all rondas campesinas in Peru. Second, although TM techniques made it possible to identify word frequencies, semantic associations, and latent topics, they cannot independently explain the motivations, internal tensions, or sociocultural meanings underlying the agreements documented in the records. During the transcription, cleaning, and tokenization of the corpus, certain discursive nuances characteristic of communal language may have been lost. Future research should therefore complement documentary analysis with interviews, observations of community assemblies, and comparative studies conducted in other regions. This would contribute to a deeper understanding of community justice, territorial governance, and legal pluralism in rural contexts.

## 5 Conclusion

The findings indicate that the priorities of the rondas campesinas vary according to the characteristics of each territory. In Huaquillas, the most prominent themes concerned development, consultation processes, and mining projects. In Namballe, illegal mining, water resources, and coordination with public authorities emerged as the principal concerns. In San José de Lourdes, assemblies and organizational strengthening were particularly salient. Across the full documentary corpus, these themes converged around development, environmental protection, community participation, and project management, demonstrating that the rondas campesinas link territorial conservation with processes of organization and collective decision-making.

The application of text-mining techniques enabled the identification of recurrent and distinctive terms, underlying thematic structures, and semantic relationships within the minutes. Word clouds, TF-IDF, LDA, and Word2Vec combined with t-SNE showed that environmental, mining-related, institutional, and organizational issues did not arise in isolation, but were interconnected within communal dynamics. These techniques proved particularly valuable for analyzing unstructured documents, identifying patterns, and achieving a more precise understanding of the concerns and forms of action developed by the rondas campesinas.

The study contributes to the sociology of law by demonstrating that the minutes not only record meetings and agreements, but also document and legitimize community-based normative practices. Through their assemblies, the rondas campesinas establish commitments, coordinate with public authorities, and organize responses to environmental, economic, and institutional problems in contexts where communal justice coexists, engages with, or comes into conflict with the state legal order. Future research could complement these findings through interviews, observations of assemblies, and comparative studies conducted in other regions, thereby deepening understanding of communal justice, territorial governance, and legal pluralism in rural areas of Peru.

## Data Availability

The original contributions presented in the study are included in the article/supplementary material, further inquiries can be directed to the corresponding author.
